# The Limitations of Hospital Training

**Published:** 1909-09-04

**Authors:** 


### THE LIMITATIONS OF HOSPITAL TRAINING.

So numerous have the sub-divisions and specialties of medicine and surgery evolved during the past generation become that it is no matter of surprise if students are sometimes a little confused during their period of study in the wards as to the exact purpose of their education and the proper proportions of certain of its component parts. While human nature remains the same, medical student nature will infallibly place first and foremost the passing of examinations; and if everything were arranged for the best in an ideal medical commonwealth such an ambition would be not only, as indeed it now is, praiseworthy, but also of the highest ultimate advantage to everyone. That is, if examinations, especially final examinations, were so arranged as to form complete tests of a man's fitness and all-round ability to enter general practice, then obviously the kind of study which best prepared him for examination would be the kind of study which should best fit him for private practice. But such a state of affairs is, in fact, extremely difficult to encompass; indeed it may be doubted whether by any examination system it ever can be brought about completely, though certainly some of the examining corporations might attempt rather more successfully than they at present do to approach towards it. Probably even the State regulation of medical qualifying examinations would fail to secure an absolutely satisfactory result in this respect.

The fact remains that in several respects final examinations as at present conducted are unsatisfactory tests of a student's fitness for private general practice; and since nine-tenths of those who enter for the qualifying examinations are destined for practice, either at home or abroad, to which the word general can rightly be applied, it may be well to consider how far the individual can rectify such a defect in his educational curriculum. To a certain extent conscious volitional effort is not of very much use. That is to say, tact, sympathy, adaptability, resource, and other somewhat vague qualities which make for success in professional life are not to be bestowed by any system of inculcation: they come naturally to some men, not at all to others, but they can be partially, at least, cultivated by keen observation. The medical student who is fortunate enough to be dresser or clerk to a hospital resident who is both devoted to his work and naturally adapted to it may learn the most valuable lessons by watching the ways in which his senior extracts information from patients, or from garrulous friends, with the maximum result and the minimum of friction and waste of time; in which he handles anxious mothers and bereaved relatives; in which he controls and yet conciliates the sisters, nurses, porters, office clerks, and other units of the hospital comity with whom he is hourly brought in contact; and in which he manages to gain the confidence and friendship of his " chief " by loyal support and intelligent prevision. House surgeons have been known who have been somewhat chaffed by their contemporaries for a supposed exaggeration of politeness in investigating the complaints of charwomen or chimney sweeps; yet even a hospital out-patient or casualty room is by no means an impossible school for the cultivation of what is called a good bedside manner; and a little extra good humour or consideration is by no means thrown away even upon the most unpromising looking of the pauper classes.

Here then is one link in the chain of professional efficiency which is never tested by examinations, and therefore often neglected by students; yet it is a link whose weakness will ruin the whole chain of carefully tested proficiency in the scientific and clinical parts of medicine. To teach these things is doubtless impossible, and for that matter not every teacher is competently outfitted himself with the qualities that make for success in general practice. A well-known London surgeon, now retired, was fond of impressing upon his dressers the aphorism " You gentlemen must remember that you will be examined by the rejected of our profession," and if this gibe contains neither the whole truth nor yet nothing but the truth, there is just enough of truth in it to make it sting. In the same way it is to a certain extent the case that those of the teachers at medical schools who are most successful in consulting practice have least time to devote to teaching students. Thus it comes to pass that, broadly speaking, the teachers are either young men whose reputation as clinicians is still in the making, or men to whom the scientific and tutorial sides of their work appeal more than the strictly utilitarian. In either case it follows that the medical student's natural inclination to study only that which will conduce directly to his success in passing examinations is somewhat apt to be encouraged at the expense of those indefinable qualities which are less marketable in an examination hall than in after life.

Then, again, since hospitals are staffed more and more by specialists, the student becomes accustomed to seeing the patients, in whose symptoms and signs he is instructed, already pigeon-holed for him into compartments. He goes into one room in an out-patient department and finds an assistant surgeon discoursing upon a series of cases all surgical in nature; or into another to find a physician dealing solely with diseases of the skin, let us say. What may easily escape his notice is that receiving-room officers or some other competent qualified functionaries have already briefly investigated these patients and assorted them, removing from the motley assemblage both those whose immediate admission seems imperative and those whose complaints are too trivial to demand regular out-patient attendance. In practice there is no such classification; cases medical, surgical, obstetrical, ophthalmological, and of many other kinds, arise haphazard, and the practitioner has to be prepared to deal with them all. To a very considerable degree the modern practitioner has to be a bit of a specialist in everything. It is true that this has always been his *rôle*, but then a couple of generations ago the conditions were hardly comparable to those that now exist.

It may be asked, how in the world can the medical student in the two years allotted to his clinical studies make himself adept in a dozen or so of specialties, besides receiving his general training and acquiring the knowledge necessary to pass his examinations? And the answer is that, very exceptional men alone excluded, he cannot do so. That this is so is not the fault of his hospital, nor of his teachers; it is a limitation caused by the inadequate period allotted to clinical studies. There is general agreement as to the method by which this limitation of hospital training may best be overcome, but unfortunately there are financial and other practical considerations which only too often prevent its realisation. The method in question is to obtain a resident hospital appointment as house surgeon, house physician, or some equivalent post. In such an office the apt and the observant will learn more of the practical application of the principles of medicine and surgery in a month than they have done in two years, or even three or four, of unqualified study. For this purpose a thorough training in the groundwork of medical education is necessary, but he who has acquired this as it is intended by licensing bodies that it should be acquired, as opposed to him who has been " crammed " through by an over-zealous " coach," will rapidly acquire a familiarity with the various sub-divisions and special departments which makes him in a year or two really a capable man in every or nearly every department of modern therapeutics.

Now this method of completing a medical education is, in a sense, an expensive one. That is, it involves the expenditure of a year or more of time in return for a salary which is often nothing at all and always very far short of the market value of the services rendered by the house officer to his hospital. Whether this state of things should or should not be rectified by the encouragement of a spirit of trades unionism among young medical men we shall not now discuss, but parents and guardians who are about to face the cost of bringing up a boy in the medical profession ought to know that after and beyond the (minimum) five years of unqualified study there comes a period during which, if he is to be fully equipped, his work will be quite unremunerative, if not actually an item of expense. A fair day's wage for a fair day's work is a principle not yet adopted in the medical profession, though signs are not wanting which indicate the possibility of its recognition, more fully than at present, in the course of a few years.

